# Growth, safety, and tolerance of a hydrolysed rice protein-based formula in infants with cow’s milk allergy

**DOI:** 10.3389/fnut.2026.1892511

**Published:** 2026-09-28

**Authors:** Malgorzata Arciszewska, Wieslaw Olechowski, Julian Lirio Casero, Stephanie Colinet, Jacqueline Cotton, Manon Oude Nijhuis, on behalf of the STORY study group

**Affiliations:** 1 Poliklinika Ginekologiczno-Poloznicza Sp. z.o.o. Sp.K, Bialystok, Poland; 2 Alergo-Med Specjalistyczna Przychodnia Lekarska Sp. z o.o., Tarnów, Poland; 3 San Rafael Hospital, Madrid, Spain; 4 CHC MontLégia, Liège, Belgium; 5 Danone Research and Innovation, Utrecht, Netherlands

**Keywords:** cow’s milk protein allergy, gastrointestinal symptoms, growth, hydrolysed rice protein-based formula, tolerance

## Abstract

**Background:**

Cow’s milk allergy (CMA) is the most common food allergy in infants, requiring the use of hypoallergenic formulas when breastfeeding is not possible. Hydrolysed rice formulas (HRF) are now positioned alongside extensively hydrolysed cow’s milk protein formulas as first line (nutritional) management of CMA in both European and International guidelines. This descriptive single-arm, multicentre interventional study (NCT05618704) evaluated growth, safety, and tolerance of a new HRF in infants with CMA.

**Methods:**

Infants ≤8 months with CMA, diagnosed by their clinicians according to the local hospital practice, were recruited from clinics in Belgium, Poland and Spain. The infants (*n* = 69) were prescribed the new HRF and followed for 16 weeks. Adequate growth was assessed using WHO growth z-scores weight-for-age and weight-for-length. Allergic symptoms (assessed by the clinician using Cow’s Milk-related Symptom Score [CoMiSS®]), gastrointestinal symptoms (reported in diaries by parents) and safety were monitored during the 16-week period. Growth and efficacy analyses were primarily conducted on infants who completed the 16-week observational period (*n* = 61), with sensitivity analyses performed in the all subjects treated (AST) population. Safety analyses were conducted on all infants that consumed the HRF (*n* = 68).

**Results:**

At baseline, mean age (±SD) was 3.17 ± 2.32 months. Over the 16-week period, increases in weight-for-age and weight-for-length were observed, with 92% of infants (56/61) meeting the predefined criteria for adequate growth. Sensitivity analyses in the AST population yielded consistent conclusions. Cow’s milk related symptoms decreased over time; mean CoMiSS declined from 11.3 ± 3.4 at baseline to 2.1 ± 1.8 at 16 weeks, with reductions observed across all CMA diagnostic categories. Compared with baseline, parents reported reductions in gastrointestinal symptoms over the observation period. The overall parental opinion was that the formula was very good. No new safety signals were identified during the 16-week study period.

**Conclusion:**

Infants with clinician-diagnosed CMA who received the new HRF for 16 weeks showed adequate growth and reductions in cow’s milk related and gastrointestinal symptoms over time. The formula was well tolerated by infants and well accepted by parents, and no new safety signals were observed during the study period. These findings support the use of HRF as a nutritional option for the dietary management of CMA, in line with current guidelines.

## 1 Introduction

Cow’s milk allergy (CMA) is one of the most common food allergies in infancy, with a prevalence ranging from 2 to 4% in children under 1 year of age (1, 2). It is characterized by immune-mediated reactions to cow’s milk proteins, which can manifest through gastrointestinal (e.g., vomiting, diarrhoea, bloody stools), dermatological (e.g., eczema, urticaria), and respiratory symptoms, and in severe cases, lead to anaphylaxis (3, 4). These symptoms often impair nutritional status and growth, underscoring the need for safe and effective dietary management strategies when breastfeeding is not feasible (5). Management requires elimination of cow’s milk proteins while ensuring adequate nutrition, typically achieved through specialized hypoallergenic formulas (5–7). Traditionally, extensively hydrolysed formulas (eHF) derived from cow’s milk proteins (casein or whey) have been considered as the first step for the diagnosis and management of CMA, with amino acid-based formulas (AAF) reserved for severe cases, such as those with anaphylaxis, eosinophilic oesophagitis, or in cases of intolerance to eHF (7, 8). However, recent international consensus guidelines from the European Society for Paediatric Gastroenterology, Hepatology and Nutrition (ESPGHAN) and Diagnosis and Rationale for Action against Cow’s Milk Allergy (DRACMA) guidelines from the World Allergy Organization (WAO) also recognize hydrolysed rice protein-based formulas (HRFs) as a suitable first-line alternative to eHF (7, 9, 10). HRFs are categorized as Foods for Special Medical Purposes (FSMP) designed for the dietary management of mild to moderate CMA. Fortified with several essential amino acids that are inherently low in rice protein, HRFs support infant and child feeding without increasing the risk of nutrient inadequacy (11). HRFs offer an advantage by expanding dietary choices for families who prefer plant-based options and for infants who do not tolerate or accept an eHF (12). This flexibility promotes patient-centred care and may improve adherence and satisfaction (13).

Unlike cow’s milk-derived eHFs, which require protein hydrolysis to reduce allergenicity, HRFs are inherently free of cow’s milk proteins and thus hypoallergenic by source. They do require hydrolysis of the rice protein, but for improved solubility and digestibility rather than reduced allergenicity (12). Several studies have evaluated HRFs in CMA management, reporting favourable outcomes on growth indices in infants with CMA, symptom resolution assessed by tools such as the Cow’s Milk-related Symptom Score (CoMiSS®), and overall safety (13–19). Continued research will help strengthen confidence in HRF as part of routine clinical practice, ensuring both symptom resolution and optimal growth in affected infants.

The STORY study evaluated growth, tolerance, and safety in infants with clinician-diagnosed CMA across multiple European centres who received a new HRF over a 16-week period. By comparing our findings with published evidence on HRF and eHF, this study aims to confirm earlier findings that HRF are a nutritionally adequate and clinically effective alternative for CMA management.

## 2 Materials and methods

### 2.1 Study design and population

This prospective, single-arm, multicentre interventional study was conducted across 11 sites in three European countries (Belgium, Poland, and Spain). The study was registered at ClinicalTrials.gov (NCT05618704) and approved by relevant Institutional Ethics Committees, with written informed consent obtained from each participant’s parent or caregiver before study entry. Enrolment took place between March 2023 and June 2024.

Eligible participants were infants aged ≤8 months diagnosed with CMA by their clinician according to local hospital practice. Infants could qualify based on a convincing clinical history of CMA with symptom resolution upon elimination of cow’s milk protein (e.g., improvement when switched to a hypoallergenic formula or when a breastfeeding mother followed a milk-free diet for ≥2 weeks), a history of a CoMiSS® > 10 prior to enrolment (indicating significant multisystem symptoms), or a positive oral food challenge to cow’s milk in the referring clinic. Exclusion criteria included low or high birth weight (weight-for-age z-score <−2 SD or > + 2 SD), prematurity <37 weeks’ gestation requiring specific premature formula at the time of study entry, any severe congenital or chronic illness that could affect growth, clinical judgment favouring AAF as first-line formula, and any known or suspected allergy to rice or any ingredient in the study product. Eligible participants were prescribed HRF for a 16-week observation period. Continued complementary breast feeding was supported.

### 2.2 Study product

The study product was a nutritionally complete, powdered infant and follow-on formula (produced by Danone Research & Innovation) containing hydrolysed rice protein supplemented with single amino acids (L-lysine, L-tryptophan, and L-isoleucine), see Table 1 for further details. The HRF also contained a prebiotic blend of non-digestible oligosaccharides, specifically, short- and long-chain fructo-oligosaccharides (scFOS/lcFOS) in a 9:1 ratio. Parents were instructed on proper preparation of the study product and were asked to feed it ad libitum to their infant, aiming for at least a minimum volume per day to ensure nutritional adequacy. As general guidance, parents were advised to target a minimum daily intake of approximately 500 mL for infants 0–6 months, 400 mL for infants 6–8 months, and 300 mL for infants older than 8 months, in line with age-based nutritional needs. Infants were also being breast fed and or consuming complementary foods as appropriate in parallel to consuming the study HRF.

**Table 1:** Nutritional composition of the study product.

Components	Unit	Amount per 100 g powder	Amount per 100 mL prepared formula
Energy	kcal	496	67
Carbohydrates	g	51.0	6.9
Protein	g	12.4	1.7
Lipids	g	26.0	3.5
scFOS/lcFOS	g	4.7	0.6

### 2.3 Endpoints and assessments

Participants were evaluated at baseline (study entry) and at follow-up visits at 4, 8, 12, and 16 weeks of study product feeding. At each scheduled visit, a comprehensive assessment was performed. The primary endpoint was growth adequacy during the intervention, as measured by the proportion of participants demonstrating adequate growth, assessed using WHO weight-for-age and weight-for-length z-scores. Weight and length were measured at each visit. Each measurement was converted to a z-score for the participant’s age (and sex) using WHO Child Growth Standards. Infants were categorized at baseline and at week 16 as either ‘normal growth status’ or ‘growth concern’ based on the weight-for-age and weight-for-length z-scores. Growth was classified as adequate or inadequate according to whether infants remained within or transitioned between normal growth and growth concern categories. In essence, infants were classified as having adequate growth if their weight-for-age and weight-for-length status stayed within or improved to normal growth status from baseline to week 16. If an infant’s growth status worsened or remained suboptimal (e.g., remained in or fell into the ‘growth concern’ category by week 16), growth was classified as inadequate (detailed criteria in Appendix A – definition of adequate/inadequate growth). Continuous growth parameters (weight-for age, weight-for length and length-for-age z-scores) were collected as supportive evidence over the 16 weeks.

Other endpoints included clinical evaluation of CMA-related symptoms using CoMiSS®. Parents were provided diaries to record gastrointestinal symptoms, study product intake and study product appreciation, including acceptance and palatability. Gastrointestinal symptoms were collected as 24-h recall at baseline (before study product intake started) and over a 7-day period during weeks 1, 2, 4, 8, 12 and 16. Additional details regarding parent-reported gastrointestinal symptoms are provided in Appendix A—Parent-reported gastrointestinal symptoms. Adverse events and serious adverse events were monitored throughout the 16-week follow- up period.

### 2.4 Statistical methodology

A total sample size of 69 participants was planned to provide 80% power to determine whether the proportion of infants with adequate growth exceeded the prespecified criterion over the 16-week observation period for at least 62.5% of infants, assuming a true adequate-growth proportion of 80%, using a one-sided significance level of 0.05 and allowing for a drop-out rate of 30%. Details of the primary statistical hypothesis used for the sample size calculation can be found in Appendix A—Sample size calculation.

The primary analysis was performed in the all subjects treated completers population (infants who completed the 16-week observation period with available growth data). Safety analyses were performed in the all subjects treated (AST) population. Continuous non-primary outcomes were summarised descriptively over time. Analyses of non-primary endpoints were primarily performed to verify that observed patterns were consistent with expectations and did not indicate potential concerns related to growth, tolerance, or safety.

Adverse events were tabulated in frequency tables by using the System Organ Class and MedDRA preferred terms. Statistical analyses were performed by using SAS Life Science Analytics Framework (LSAF) version 9.4 (SAS Institute, Cary, NC).

## 3 Results

A total of 69 infants diagnosed with CMA (as determined by the recruiting clinician) were enrolled in the study. One infant, who was predominantly breastfed (approximately 90% of feeding) and receiving eHF prior to enrolment, was classified as an early withdrawal because the parents continued both feedings and never initiated intake of the study product. The remaining 68 infants did start study product intake and were included in the AST population. Among the 68 infants in the AST population, eight additional early withdrawals occurred: three due to adverse events and five at the request of the parent. Three infants with early withdrawal-related adverse events reported mild constipation (*n* = 1), gastroesophageal reflux disease (GERD) (*n* = 1), and constipation and abdominal colic (*n* = 1). The events were reported as not related (GERD) and possibly related to study product. For 61 infants, data were collected through 16 weeks, forming the AST-completers population. Participant disposition is presented in Supplementary Figure S1.

Participant demographics and baseline characteristics for the AST population are summarized in Table 2. The mean age at baseline was 3.17 (SD ± 2.32) months. Overall, 46% of participants were female, and 54% were delivered by caesarean section. CMA diagnosis by the infant’s clinician was according to local hospital practice. Regarding use of hypoallergenic formula, at study entry, 60 infants (88%) were already receiving a hypoallergenic formula: 70% of participants were taking eHF, 15% HRF, and 3% AAF. Further details on previous interventions are not available. Demographic and baseline clinical characteristics of AST-completers (*n* = 61) and non-completers (*n* = 7) are presented in Supplementary Table S1. Overall, the distributions of demographic characteristics, CMA diagnostic categories, feeding type at study entry, and baseline growth parameters were broadly comparable between completers and non-completers, although the small number of non-completers limits formal interpretation.

**Table 2:** Demographics and participant characteristics of the AST population at baseline.

Characteristic	Total (n = 68)
Age at baseline (months), mean ± SD	3.17 ± 2.32
Sex, *n* (%)	
Male	37 (54)
Female	31 (46)
Mode of delivery, *n* (%)	
Caesarean section	37 (54)
Vaginal	31 (46)
Country of residence, *n* (%)	
Belgium	7 (10)
Poland	57 (84)
Spain	4 (6)
CMA diagnosis, *n* (%)	
Symptom resolution following cow’s milk elimination	18 (26)
History of CoMiSS >10	33 (49)
Combination of symptom resolution and CoMiSS >10	12 (18)
Positive open food challenge	5 (7)
Type of feeding at study entry, *n* (%)	
Exclusively breastfed	8 (12)
eHF	48 (70)
AAF	2 (3)
HRF	10 (15)

Denominator for percentage is number of participants with non-missing data (*n* = 68). SD, standard deviation, n, number of participants.

Among the 61 infants that completed the study, 56 (91.8%) met the pre-defined criteria for adequate growth, while 8.2% (*n* = 5) did not. The proportion of adequate growth was 0.918 with an exact one-sided 95% lower confidence bound of 0.8354 (AST-completers population). Since this bound exceeds 0.625, the primary objective was met. As an additional sensitivity analysis, participants without a week 16 anthropometric assessment were conservatively classified as not having achieved adequate growth. The conclusions were unchanged, with the lower bound of the confidence interval remaining above the predefined threshold. Notably, for the 5 infants who fell into the ‘inadequate growth’ category by the study’s pre-defined definition after 16 weeks of intervention, their clinicians had no concerns about their growth; in fact, all infants either stayed on their expected growth curves or improved in weight-for-age and weight-for-length percentiles. In other words, the few infants flagged by the strict criteria were still growing normally according to clinical judgment.

Analysis of continuous growth data over time showed that for infants who received the HRF and completed follow-up, growth trajectories remained within normal ranges (weight-for-age, length-for-age, and weight-for-length mean z-scores were in the range −1.0 SD to +1.0 SD over 16 weeks) and weight parameters increased over time, consistent with mild catch-up growth (Figure 1). Similar findings were observed in the AST population (data not shown).

**Figure 1:**
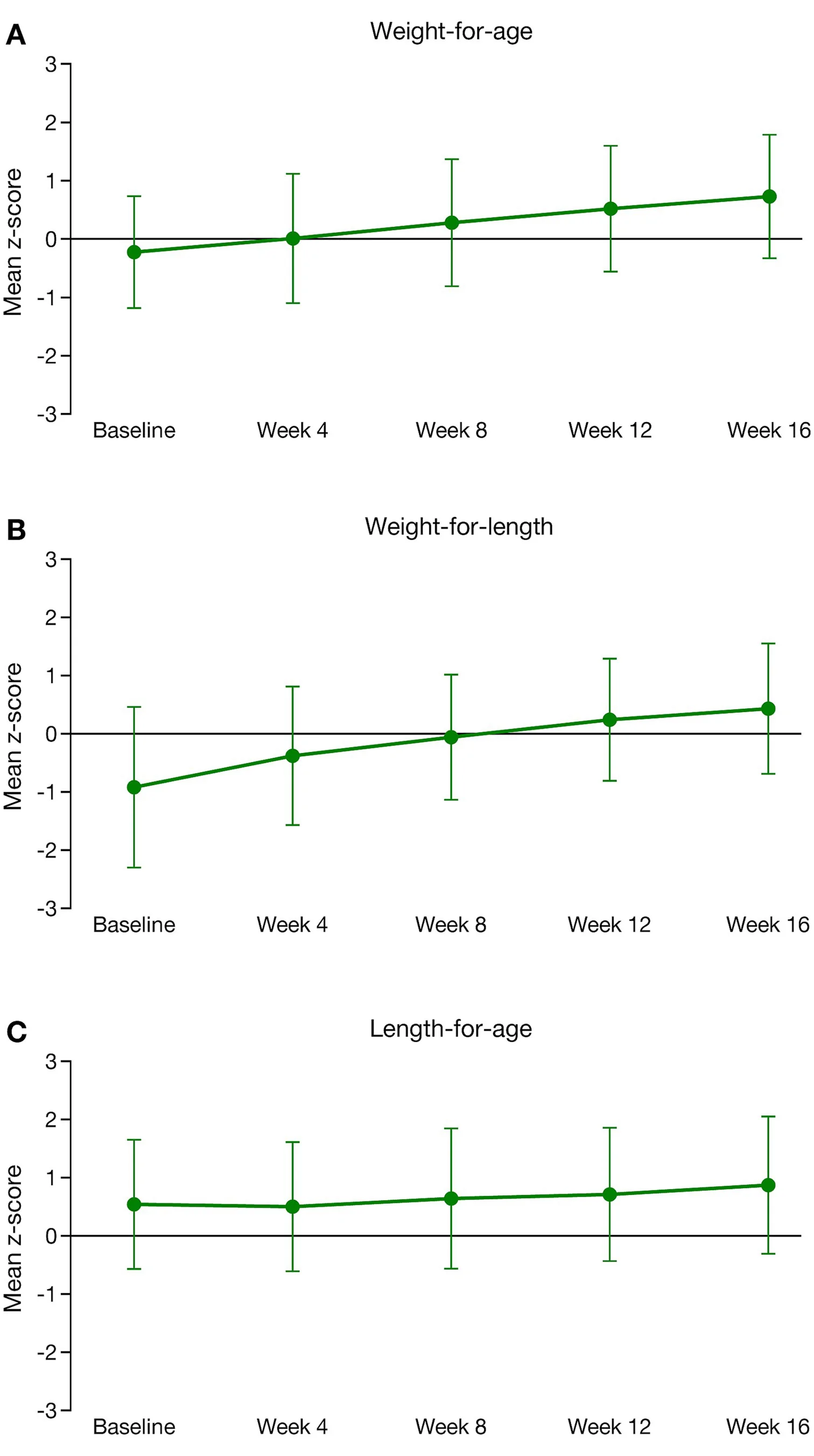
Trajectories of mean anthropometric `*z*`-scores. Mean weight-for-age `*z*`-score **(A)**, weight-for-length `*z*`-score **(B)**, and length-for-age `*z*`-score **(C)** measured at baseline and at follow-up visits at 4, 8, 12, and 16 weeks of study product feeding in AST-completers. Line at `*z*` = 0 represents the WHO standard median. Data are presented as mean ± SD.

During the 16-week observation with the HRF, the total CoMiSS decreased over time (Table 3). The mean ± SD CoMiSS decreased from 11.3 ± 3.4 at baseline to 2.1 ± 1.8 by week 16, reflecting reduction in cow’s milk related symptoms. The proportion of participants with CoMiSS ≥10, indicative of significant symptom burden, dropped from 80.3% (49/61) at baseline, to 13.1% (8/61) within 8 weeks and 0% within 12 weeks of HRF intake. Similar findings were observed in the AST population. When CoMiSS data were stratified according to CMA diagnostic category, reductions in total CoMiSS score from baseline to week 16 were observed, indicating improvement in cow’s milk related symptoms across all diagnostic categories included in the study (Supplementary Table S2). All symptom domains included in CoMiSS such as crying, regurgitation, stool consistency, skin, and respiratory symptoms, showed improvement, with a higher proportion of infants being symptom-free or in the least severe category by 16 weeks (Supplementary Figure S2).

**Table 3:** Cow’s milk-related symptom score (CoMiSS) for the AST-completers population.

Time point	CoMiSS (mean ± SD)	Participants with CoMiSS ≥10 n (%)
Baseline	11.3 ± 3.4	49 (80)
4 weeks	7.3 ± 4.2	29 (48)
8 weeks	5.5 ± 3.5	8 (13)
12 weeks	3.5 ± 2.1	0 (0)
16 weeks	2.1 ± 1.8	0 (0)

Denominator for percentage is number of participants with non-missing data (*n* = 61). CoMiSS, Cow’s Milk-related Symptom Score; SD, standard deviation; n, number of participants.

Severity of parent-reported gastrointestinal symptoms decreased over the 16-week observation period. At baseline, moderate-to-severe regurgitation was common, whereas by week 16, most infants were free of significant regurgitation. Abdominal pain followed a similar pattern: while many infants initially experienced moderate or severe symptoms, none reported more than mild abdominal pain by week 16, and the majority were symptom-free. Likewise, episodes of diarrhoea and constipation were rare or mild by the end of the study, in contrast to a few moderate cases observed at baseline. Figure 2 illustrates the overall shift from moderate or severe symptoms at baseline to predominantly mild or absent symptoms across all categories by week 16 (AST-completers population). Findings in the AST population were consistent with those observed in the AST-completers population (data not shown).

**Figure 2:**
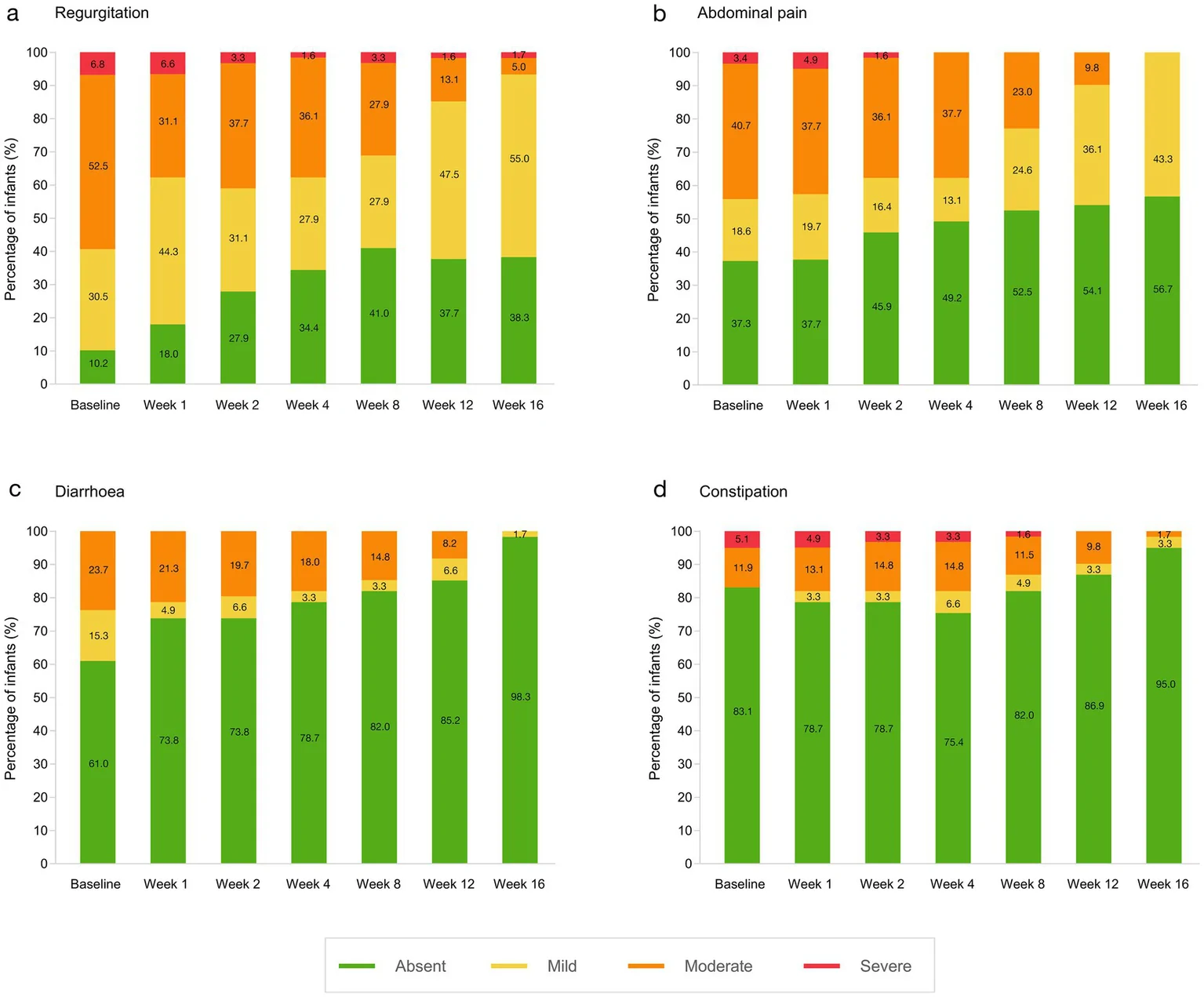
Parent-reported gastrointestinal symptoms. Percentage of participants (AST-completers) with absence or presence of mild, moderate or severe gastrointestinal symptoms at baseline and during weeks 1, 2, 4, 8, 12 and 16 of study product feeding. Median levels of severity over 7-day period were collected for **(a)** Regurgitation, **(b)** Abdominal pain, **(c)** Diarrhoea, and **(d)** Constipation. In case of even measurement, fractional numbers have been rounded up to the higher integer.

The study product was generally well accepted by the infants. In the AST-completers population, average study product intake per day ranged from 764 ± 201 mL to 790 ± 192 mL. These volumes are in line with expected infant requirements and indicate good compliance. At the end of the observation period, 97% of the parents agreed or strongly agreed that their child liked the taste of the study product, and that the study product was gentle on their child’s tummy. All parents agreed or strongly agreed that their child adapted well to the study product, and overall liking was rated as very good.

None of the infants experienced any acute allergic reaction to the study product (no hives, wheezing or anaphylaxis upon introduction of the study product). Also, no cases of rice protein allergy were detected. During the 16-week observation period, 38% of the infants experienced at least one adverse event, the majority of which were assessed as unrelated to the study product. An overview table of adverse events is provided in Supplementary Table S3. Most events were categorized as gastrointestinal disorders or infections/infestations, such as common childhood illnesses like colds. As noted above, three infants withdrew due to adverse events (constipation/colic or GERD). Three serious adverse events (SAE) were reported (mild COVID-19, a severe lower respiratory tract infection, and a moderate acute respiratory syncytial virus bronchiolitis), occurring in three infants. All three infants were hospitalised and recovered, and the SAEs were considered not related to the study product. Importantly, no new safety signals were identified during the 16-week observation period.

## 4 Discussion

This study evaluated growth, safety and tolerance of a new HRF with added prebiotics in infants diagnosed with CMA. In this single-arm, open-label observational study, infants were diagnosed according to local hospital practice which led to a potentially heterogenous group of participants. In spite of this, over a 16-week observation with the new HRF, adequate growth was demonstrated with WHO weight-for-age and weight-for-length z-scores remaining within normal ranges. The study focused on weight-for-age and weight-for-length z-scores as the key indicators of growth status, since these capture overall weight gain and proportional growth, respectively. The categorical approach for primary outcome (adequate vs. inadequate growth) was chosen to mirror clinical assessment of thriving, and a similar framework of assessing nutritional status based on growth indicators was employed by Setiabudiawan et al., who reported statistically significant differences between baseline and 6-month weight-for-age, length-for-age, weight-for-length, and head circumference-for-age nutritional status of infants with CMA fed with soy-based infant formula (20). Of the 61 infants evaluated in the current study, 56 infants demonstrated adequate growth, while only 5 did not meet the predefined growth criteria. The estimated proportion of adequate growth was 0.918 (one-sided 95% CI: 0.8354–1.0000), indicating that most infants met the prespecified growth adequacy criterion. For the five infants that did not meet the predefined criteria, their clinicians indicated that there were no clinical concerns regarding their growth, and these infants were developing normally. In addition, maintenance of continuous weight-for-age, length-for-age, and weight-for-length z-scores within normal WHO reference ranges throughout the study suggests nutritional adequacy of the HRF.

HRFs have shown the ability to support normal growth in infants and children with CMA. Previous HRF trials in infants with CMA fed HRF for 6 months or even up to 2 years of age reported no significant differences in anthropometric measures between HRF, soy formula and eHF groups over 12 months and showed normal growth patterns (13, 21, 22). Similarly, earlier single-arm HRF studies confirmed normal growth trajectories in infants with CMA fed HRF (14, 16). Interestingly, Vandenplas et al. noted that some infants in their study, who had shown poor growth on regular formula prior to diagnosis, demonstrated catch-up growth after switching to HRF, with normalization of weight-for-age percentiles over the 6 months of treatment (16). It is worth noting that the group of infants in this study had relatively low weight-for-length z-scores at baseline, which may reflect the nutritional consequences of CMA, delayed diagnosis and or inadequate nutritional intake. As in the study by Vandenplas and colleagues, increases in the weight-for-length z-scores were seen over the 16 weeks in this study.

Regarding cow’s milk-related symptoms, lower CoMiSS values were observed over time, from a baseline mean of 11.3 to 2.1 at week 16, with 100% of infants achieving scores <10 by week 12. By week 16, a higher proportion of infants were either symptom-free or classified within the least severe category for each symptom domain, confirming the broad clinical benefit of HRF in managing CMA-related manifestations. These findings are consistent with clinically relevant reductions in cow’s milk-related symptom burden over time during HRF use. In addition to using the CoMiSS as a standardized measure of CMA symptoms, we captured more granular gastrointestinal tolerance parameters in daily parent diaries. Gastrointestinal symptoms were also monitored as part of the safety review. Overall, gastrointestinal tolerance outcomes were favourable during follow-up. A few infants had transient changes in gastrointestinal symptoms in the first days after switching to the HRF - a commonly reported effect when changing to any new formula. After the initial adaptation period, there were no reports of ongoing feeding intolerance; in fact, gastrointestinal symptoms generally improved markedly during the follow up period. Throughout the intervention, parents agreed that their child seemed to like the taste of the HRF, to adapt well to the formula and that the HRF seemed to be gentle on their child’s tummy. Acceptability and palatability are important aspects, especially to prevent refusal of hypoallergenic formulas. Several studies reported palatability and/or acceptability of HRF to be good, even though some authors also report refusal of the HRF in their studies (15, 16, 23, 24).

The marked reduction in CoMiSS and decrease in parent-reported gastrointestinal symptoms observed over time are consistent with HRF being a suitable nutritional option for infants with CMA, in line with current management guidelines. Vandenplas et al. documented a significant decrease in symptom-based allergy score within the first month of HRF use, accompanied by improvements in stool consistency and reductions in colic and regurgitation. By 6 months of treatment, most infants with CMA had minimal residual symptoms (16, 25). The authors concluded the HRF was tolerated by more than 90% of children with proven CMA (95% CI). Lemoine et al. investigated tolerance acquisition in infants with food challenge-confirmed CMA and observed a non-statistically significant trend towards faster acquisition of tolerance in the HRF group compared to the eHF group (median 20.4 versus 16.3 months) (13). Similarly, Reche et al. evaluated the longer-term course of CMA and found that the proportion of infants who outgrew their milk allergy by 12–24 months of age was comparable between those managed with HRF and those reported in literature on eHF, with over half of the infants tolerating cow’s milk by 2 years of age (15). Infants receiving HRF did not experience vomiting or other digestive symptoms attributable to the formula, and no infants discontinued treatment due to gastrointestinal intolerance. These findings suggest that the use of HRF as an elimination diet does not impede the natural development of tolerance to cow’s milk protein. A systematic review by Saad et al. encompassing 17 studies and 1,695 infants with CMA, confirmed that HRFs effectively manage CMA, with allergic symptoms consistently improving and symptom-based scores decreasing over time, typically within the first 1–6 months of intervention (26). In addition, Ramirez-Farias et al. demonstrated that a four-week intervention with HRF supplemented with 2′-fucosyllactose (2′-FL) in infants with suspected protein allergies (CMA and/or soy) resulted in significant improvements in regurgitation/vomiting, stool consistency and other gastrointestinal symptoms (17).

Eligible participants were infants ≤8 months of age who were diagnosed with CMA by their clinician. The diagnostic criteria were pragmatic and mirrored routine clinical practice, to broaden applicability. This approach increases generalizability, though it relies on clinical judgment for diagnosis; therefore, some heterogeneity in diagnostic work-up across sites cannot be excluded. The inclusion of young infants, including those who used HRF as sole source of nutrition, together with the encouraging findings of high HRF intake and positive parental acceptance observed in our study, suggest that HRF may be used as the sole source of nutrition in infants similar to those included in this study. This is supported by the favourable safety profile observed in this study, with no cases of rice protein allergy and only mild-to-moderate gastrointestinal adverse events during product transition. Published HRF trials similarly report no infants with allergic reactions to the HRF (13–15). The pattern of adverse events (mainly common infant illnesses) was consistent with what would be expected in an infant population, and no new safety signals were identified during the 16-week follow up period.

Prebiotics have been added to the HRF with the aim to potentially beneficially affect the gut microbiota of the infants. Prebiotic oligosaccharides are non-digestible food ingredients that beneficially affect the host by selectively stimulating the growth and/or activity of one or a limited number of bacteria in the colon that can improve host health (27). Infants given oligofructose-supplemented formula tend to develop a colonic microbiota that more closely resembles that found in breastfed infants compared with infants fed the same formula without oligofructose (28, 29). Additionally, infant formula containing oligofructose produces more frequent and softer stools than does control formula, more closely matching the stool characteristics of breastfed infants (28–30). Together these findings likely indicate that the addition of functional ingredients such as oligosaccharides to HRF is feasible and does not compromise tolerance or growth, consistent with the observations from our study.

Limitations of the study include its descriptive single-arm design and relatively short duration. Without a comparator group, care also needs to be taken with the interpretation of the results. Although only a small proportion of participants discontinued the study and no major baseline imbalances were apparent between completers and non-completers, the potential for attrition bias cannot be fully excluded. Furthermore, participants were included based on different CMA diagnostic pathways, ranging from symptom-based clinical assessment to confirmation by open food challenge. As these diagnostic categories may represent different levels of diagnostic certainty and baseline symptom severity, subgroup-specific findings should be interpreted with caution. Nevertheless, reductions in CoMiSS were observed across all diagnostic categories, supporting the consistency of the overall symptom improvement observed during the study. The findings of the present study seem to be consistent with other studies investigating the effects of HRF in infants with CMA, but it should be noted that the findings from this study are only applicable to similar populations. The effects of HRF in patients with anaphylaxis or more complex diagnoses were not described in this study. Even though in this study the HRF was not directly compared with other dietary interventions, the results of the study potentially contribute to the growing body of evidence supporting HRF as a nutritionally adequate, clinically effective, and well-tolerated alternative to eHF for managing CMA. HRF may offer additional benefits, including plant-based positioning, which may appeal to parents seeking non-dairy options. These findings align with international guidelines that recognize HRF as an acceptable choice for CMA management when appropriately formulated.

## 5 Conclusion

In this study, infants with clinician-diagnosed CMA who received an HRF with added prebiotics maintained adequate growth over the 16-week observation period, supported by both the predefined growth endpoint and continuous growth parameters. Reductions in cow’s milk-related symptom scores were observed over time. The formula was generally well tolerated, parental feedback indicated good acceptance, and no new safety signals were observed during the study period. These findings support HRF as a nutritional management option for infants with mild-to-moderate CMA and a clinical profile similar to that of the infants included in this study. While future controlled trials could further strengthen the evidence base, our findings add to the growing body of evidence supporting the use of HRF in the dietary management of CMA.

## Data Availability

The data presented in this study are available on request from the corresponding author due to privacy and ethical restrictions related to the protection of participants’ personal data. Requests to access the datasets should be directed to manon.oude-nijhuis@danone.com.

## Group member of the STORY study group

STORY study group includes: Marzena Nowak (Promed Medical Centre, Kraków, Poland; marzenanowak@interia.com), Malgorzata Bederska (Malopolskie Centrum Alergologii sp.z o.o., Kraków, Poland; mbederska@icloud.com), Monika Konwinska (EPOKA Niepubliczny Zaklad Opieki Zdrowotnej Piotr Chodkiewicz Sp. z o.o, Żnin, Poland; monikakonwinska@wp.pl), Els Ide (General Hospital Delta, Roeselare, Belgium; els.ide@azdelta.be), and Koen Kaïret (General Hospital Voorkempen, Malle, Belgium; koen.kairet@emmaus.be).
