# Supplementary material for: Growth, safety, and tolerance of a hydrolysed rice protein-based formula in infants with cow’s milk allergy
Source: Front Nutr. 2026 Sep 28;13:1892511. doi: 10.3389/fnut.2026.1892511 (PMC13656850; doi:10.3389/fnut.2026.1892511)
Supplement: Supplementary file 1 [file Supplementary_file_1.docx]

Supplementary Material

# 1 Supplementary Data

**Definition of adequate / inadequate growth**

For the purposes of the study, growth was classified according to WHO weight-for-age and weight-for-length z scores [31]. These scores were used to put the participants in categories including a normal category (normal growth status – weight-for-age and weight-for-length z-scores between -2SD and +1SD) or growth problem category (growth concern) - being severely underweight (weight-for-age z-score <-3SD), underweight (weight-for-age z-score <-2SD), possible risk of overweight (weight-for-length z-score between +1SD and +2SD), overweight (weight-for-length z-score >+2SD) or obese (weight-for-length z-score >+3SD) - at baseline and at the end of the 16-week study period. Growth was classified as adequate only if infants were in the normal range at baseline and at 16 weeks, moving from normal range at baseline to possible risk of overweight at 16 weeks, moving from possible risk of overweight at baseline to normal range at 16 weeks, moving from underweight at baseline to normal range at 16 weeks, or moving from severely underweight at baseline to underweight or to normal range at 16 weeks. The move to ‘possible risk of overweight’ (weight-for-length z-score between +1SD and +2SD) will be considered adequate because the WHO definition of overweight for children under 5 years of age is ‘weight-for-height greater than 2 standard deviations above WHO Child Growth Standards median’. WHO experts have indicated to cautiously approach overweight because young children are still growing. A mild weight increase in a rapidly growing infant is usually not harmful and the risk that young children will be placed on restrictive diets due to classification of overweight should be avoided [32]. For all other infants, whose growth did not follow these specific growth patterns, growth was classified as inadequate for the primary analyses. Using these definitions infants who did follow their growth trajectories from baseline to 16 weeks could be classified as having inadequate growth. In those cases, the treating clinician’s opinion about the infant’s growth was checked as well.

**Parent-reported gastrointestinal symptoms**
Gastrointestinal symptoms were collected by parents / guardians in a daily diary as 24-hr recall at baseline (before study product intake started) and over a 7-day period during weeks 1, 2, 4, 8, 12 and 16. At each visit, diary entries were discussed with the parents / guardians by the Investigator to ensure that the diary information was complete, correct, and verified for plausibility.

Gastrointestinal symptoms included abdominal discomfort or pain, bloating, burping, flatulence, regurgitation / spitting up / vomiting milk, nausea, vomiting, diarrhoea and constipation.

More explanation of the gastrointestinal symptoms was provided to the parents / guardians to facilitate rating. The following guidance was given for each of the gastrointestinal symptoms: Bloating: swollen tummy;

- Flatulence: passing wind;
- Regurgitation / spitting up / vomiting milk: when milk comes out of your child’s mouth, often right after feeding. Do not include milk out of your child’s mouth after burping. Do not include vomiting when your child is being sick;
- Nausea: Feeling sick / retching
- Vomiting (being sick): Forceful ejection of the contents of the stomach through the mouth and sometimes through the nose of your child.
- Diarrhoea: 3 or more watery stools per day
- Constipation: Less than 3 bowel movements per week

Parents indicated per day whether the gastrointestinal symptoms were absent or present and if present, rated its severity. Severity of the symptoms was rated on a four-point scale as: 0 (absent), 1 (mild), 2 (moderate), or 3 (severe). For each symptom, the change from ‘usual’ (before the participant entered the study) was to be rated as: 1 (better), 2 (worse), or 3 (no change).

For analyses, the median severity over each 7-day period was summarized. When an even number of observations resulted in a value between two severity categories, the higher severity category was assigned. Analyses were based on available observations only. For each assessment period, summaries were calculated using available diary entries, and both non-missing and missing observations were reported in summary tables.

**Sample size calculation**

The primary endpoint was the proportion of infants classified as having adequate growth at week 16 according to the pre‑specified WHO weight‑for‑age and weight‑for‑length z‑score–based criteria. The primary statistical hypothesis was formulated as:

H0: p ≤ 0.625 versus H1: p > 0.625,

Where *p* denotes the proportion of infants with adequate growth. The proportion p was estimated together with an exact (Clopper–Pearson) one‑sided 95% confidence bound based on the binomial distribution. The primary objective was considered met if the lower one‑sided 95% confidence bound exceeded 0.625.

The threshold of 62.5% (5 out of 8 infants) was prespecified during study planning as a pragmatic acceptability criterion. Given the wide age range of enrolled infants and the resulting heterogeneity in expected growth trajectories, it was not feasible to define a single clinically meaningful absolute change in anthropometric measures applicable across all participants. Therefore, individual growth trajectories were classified as adequate or inadequate based on WHO standards, and the primary objective was formulated in terms of the proportion of infants demonstrating adequate growth over 16 weeks of intervention.

# 2 Supplementary Figures and Tables

## 2.1 Supplementary Figures

**Supplementary Figure S1.** **Participant flow chart.** Flow chart showing the flow of participants in the study. AST = all subjects treated. Three participants with early withdrawal-related adverse events reported mild constipation (n=1), gastroesophageal reflux disease (GERD) (n=1), and constipation and abdominal colic (n=1). The events were reported as not related (GERD) and possibly related to study product.

**Supplementary Figure S2. Cow’s Milk-related Symptom Score (CoMiSS) symptom domains.** Percentage of participants in each category for a) crying, b) regurgitation, c) stools (BITSS), and d) atopic eczema in head, neck and trunk collected as part of the CoMiSS tool at baseline and at follow-up visits at 4, 8, 12, and 16 weeks of study product feeding in AST-completers. BITSS = Brussels Infant and Toddler Stool Scale.

**Supplementary Table S1. Demographics and participant characteristics of the AST-completers and non-completers at baseline**

| Characteristic | Completers (n=61) | Non-completers (n=7) |
| --- | --- | --- |
| Age at baseline (months), mean ± SD | 3.17 ± 2.39 | 3.19 ± 1.63 |
| Sex, n (%) |  |  |
| Male | 35 (57) | 2 (29) |
| Female | 26 (43) | 5 (71) |
| Mode of delivery, n (%) |  |  |
| Caesarean section | 32 (53) | 5 (71) |
| Vaginal | 29 (48) | 2 (29) |
| Country of residence, n (%) |  |  |
| Belgium | 7 (12) | 0 (0) |
| Poland | 51 (84) | 6 (86) |
| Spain | 3 (5) | 1 (14) |
| CMA diagnosis, n (%) |  |  |
| Symptom resolution following cow’s milk elimination | 16 (26) | 2 (29) |
| History of CoMiSS >10 | 31 (51) | 2 (29) |
| Combination of symptom resolution and CoMiSS >10 | 10 (16) | 2 (29) |
| Positive open food challenge | 4 (7) | 1 (14) |
| Type of feeding at study entry, n (%) |  |  |
| Human milk | 8 (13) | 0 (0) |
| eHF | 42 (69) | 6 (86) |
| AAF | 2 (3) | 0 (0) |
| HRF | 9 (15) | 1 (14) |
| Weight-for-age at baseline (WHO z-score), mean ± SD | -0.224 ± 0.957 | -0.607 ± 0.296 |
| Weight-for-length at baseline (WHO z-score), mean ± SD | -0.920 ± 1.384 | -1.215 ± 0.683 |

Denominator for percentage is number of participants with non-missing data (n=7 and n=61, respectively). SD = standard deviation, n = number of participants

**Supplementary Table S2: Cow’s Milk-related Symptom Score (CoMiSS), by Cow’s Milk Allergy diagnosis category for the AST population**

| CMA diagnosis | Baseline | 16 weeks | Change from baseline |
| --- | --- | --- | --- |
|  | mean ± SD | mean ± SD  (nmiss) | mean ± SD  (nmiss) |
| Symptom resolution following cow's milk elimination (n=18) | 7.5 ± 4.2 | 1.8 ± 2.1 (2) | -5.6 ± 5.4 (2) |
| History of CoMISS > 10 (n=33) | 12.2 ± 1.8 | 2.4 ± 1.4 (2) | -10.1 ± 2.2 (2) |
| Combination of symptom resolution and CoMiSS >10 (n=12) | 13.2 ± 1.5 | 2.0 ± 2.4 (2) | -11.2 ± 2.9 (2) |
| Positive open food challenge (n=5) | 12.8 ± 1.3 | 1.5 ± 2.4 (1) | -11.3 ± 2.1 (1) |

CoMiSS = Cow’s Milk-related Symptom Score, SD = standard deviation, n = number of participants, nmiss = number of participants with missing observation

**Supplementary Table S3. Overview of adverse events in the AST population (n=68)**

| **Category** | **n (%)** |
| --- | --- |
| **Number of participants with at least one adverse event** | **26 (38.2%)** |
| Gastrointestinal disorders^1^ | 16 (23.5%) |
| Infections and infestations^1^ | 16 (23.5%) |
| **Number of participants with at least one serious adverse event^2^** | **3 (4.4%)** |
| COVID-19 (mild) | 1 (1.5%) |
| Lower respiratory tract infection (severe) | 1 (1.5%) |
| Acute respiratory syncytial virus bronchiolitis (moderate) | 1 (1.5%) |
| **Number of participants with at least one adverse event related to the study product^3^** | **9 (13.2%)** |
| Gastrointestinal disorders | 8 (11.8%) |
| **Number of participants with adverse event leading to early withdrawal** | **3 (4.4%)** |
| Mild constipation | 1 (1.5%) |
| Gastroesophageal reflux disease (GERD) | 1 (1.5%) |
| Constipation and abdominal colic | 1 (1.5%) |

AST = all subjects treated. AE = adverse event. SAE = serious adverse event. n = number of participants, % = percentage of participants related to AST population

^1^The 2 most frequent reported system organ classes are listed.

^2^All three infants were hospitalized and recovered, and the SAEs were considered not related to the study product.

^3^Related AEs were defined as AEs for which the Investigator indicated the relationship to the study product as definite, probable, or possible. There was a total of 14 events of related AEs reported in 9 participants. Most of these AEs were in the SOC gastrointestinal disorders (12 events in 8 subjects). The other two related AEs were “trouble feeding” (decreased appetite happening concurrently with the gastrointestinal AE) and anemia.

**References**

31.	**World Health Organization. Training Course on Child Growth Assessment. Geneva, WHO, 2008.** [https://www.who.int/publications/i/item/9789241595070]

32.	de Onis M, Lobstein T: **Defining obesity risk status in the general childhood population: which cut-offs should we use?** *Int J Pediatr Obes* 2010, **5:**458–460. doi: 10.3109/17477161003615583
